# Detecting lesion-specific ischemia in patients with coronary artery disease with computed tomography fractional flow reserve measured at different sites

**DOI:** 10.1186/s12880-023-01031-4

**Published:** 2023-06-06

**Authors:** Zhaoxi Cai, Taihui Yu, Zehong Yang, Huijun Hu, Yongqing Lin, Haifeng Zhang, Meiwei Chen, Guangzi Shi, Jun Shen

**Affiliations:** 1 Department of Radiology, Sun Yat-Sen Memorial Hospital, Sun Yat-Sen University, Guangzhou, China; 2 Department of Cardiology, Sun Yat-Sen Memorial Hospital, Sun Yat-Sen University, Guangzhou, China

**Keywords:** Coronary artery disease, Computed tomography angiography, Fractional flow reserve

## Abstract

**Objectives:**

Whether a stenosis can cause hemodynamic lesion-specific ischemia is critical for the treatment decision in patients with coronary artery disease (CAD). Based on coronary computed tomography angiography (CCTA), CT fractional flow reserve (FFR_CT_) can be used to assess lesion-specific ischemia. The selection of an appropriate site along the coronary artery tree is vital for measuring FFR_CT_. However the optimal site to measure FFR_CT_ for a target stenosis remains to be adequately determined. The purpose of this study was to determine the optimal site to measure FFR_CT_ for a target lesion in detecting lesion-specific ischemia in CAD patients by evaluating the performance of FFR_CT_ measured at different sites distal to the target lesion in detecting lesion-specific ischemia with FFR measured with invasive coronary angiography (ICA) as reference standard.

**Methods:**

In this single-center retrospective cohort study, a total of 401 patients suspected of having CAD underwent invasive ICA and FFR between March 2017 and December 2021 were identified. 52 patients having both CCTA and invasive FFR within 90 days were enrolled. Patients with vessels 30%-90% diameter stenosis as determined by ICA were referred to invasive FFR evaluation, which was performed 2–3 cm distal to the stenosis under the condition of hyperemia. For each vessel with 30%–90% diameter stenosis, if only one stenosis was present, this stenosis was selected as the target lesion; if serial stenoses were present, the stenosis most distal to the vessel end was chosen as the target lesion. FFR_CT_ was measured at four sites: 1 cm, 2 cm, and 3 cm distal to the lower border of the target lesion (FFR_CT_-1 cm, FFR_CT_-2 cm, FFR_CT_-3 cm), and the lowest FFR_CT_ at the distal vessel tip (FFR_CT_-lowest). The normality of quantitative data was assessed using the Shapiro–Wilk test. Pearson's correlation analysis and Bland–Altman plots were used for assessing the correlation and difference between invasive FFR and FFR_CT_. Correlation coefficients derived from Chi-suqare test were used to assess the correlation between invasive FFR and the cominbaiton of FFR_CT_ measred at four sites. The performances of significant obstruction stenosis (diameter stenosis ≥ 50%) at CCTA and FFR_CT_ measured at the four sites and their combinations in diagnosing lesion-specific ischemia were evaluated by receiver-operating characteristic (ROC) curves using invasive FFR as the reference standard. The areas under ROC curves (AUCs) of CCTA and FFR_CT_ were compared by the DeLong test.

**Results:**

A total of 72 coronary arteries in 52 patients were included for analysis. Twenty-five vessels (34.7%) had lesion-specific ischemia detected by invasive FFR and 47 vesseles (65.3%) had no lesion-spefifice ischemia. Good correlation was found between invasive FFR and FFR_CT_-2 cm and FFR_CT_-3 cm (*r* = 0.80, 95% CI, 0.70 to 0.87,* p* < 0.001; *r* = 0.82, 95% CI, 0.72 to 0.88, *p* < 0.001). Moderate correlation was found between invasive FFR and FFR_CT_-1 cm and FFR_CT_-lowest (*r* = 0.77, 95% CI, 0.65 to 0.85,* p* < 0.001; *r* = 0.78, 95% CI, 0.67 to 0.86, *p* < 0.001). FFR_CT_-1 cm + FFR_CT_-2 cm, FFR_CT_-2 cm + FFR_CT_-3 cm, FFR_CT_-3 cm + FFR_CT_-lowest, FFR_CT_-1 cm + FFR_CT_-2 cm + FFR_CT_-3 cm, and FFR_CT_-2 cm + FFR_CT_-3 cm + FFR_CT_-lowest were correatled with invasive FFR (*r* = 0.722; 0.722; 0.701; 0.722; and 0.722, respectively; *p* < 0.001 for all). Bland–Altman plots revealed a mild difference between invasive FFR and the four FFR_CT_ (invasive FFR vs. FFR_CT_-1 cm, mean difference -0.0158, 95% limits of agreement: -0.1475 to 0.1159; invasive FFR vs. FFR_CT_-2 cm, mean difference 0.0001, 95% limits of agreement: -0.1222 to 0.1220; invasive FFR vs. FFR_CT_-3 cm, mean difference 0.0117, 95% limits of agreement: -0.1085 to 0.1318; and invasive FFR vs. FFR_CT_-lowest, mean difference 0.0343, 95% limits of agreement: -0.1033 to 0.1720). AUCs of CCTA, FFR_CT_-1 cm, FFR_CT_-2 cm, FFR_CT_-3 cm, and FFR_CT_-lowest in detecting lesion-specific ischemia were 0.578, 0.768, 0.857, 0.856 and 0.770, respectively. All FFR_CT_ had a higher AUC than CCTA (all* p* < 0.05), FFR_CT_-2 cm achieved the highest AUC at 0.857. The AUCs of FFR_CT_-2 cm and FFR_CT_-3 cm were comparable (*p* > 0.05). The AUCs were similar between FFR_CT_-1 cm + FFR_CT_-2 cm, FFR_CT_-3 cm + FFR_CT_-lowest and FFR_CT_-2 cm alone (AUC = 0.857, 0.857, 0.857, respectively; *p* > 0.05 for all). The AUCs of FFR_CT_-2 cm + FFR_CT_-3 cm, FFR_CT_-1 cm + FFR_CT_-2 cm + FFR_CT_-3 cm, FFR_CT_-and 2 cm + FFR_CT_-3 cm + FFR_CT_-lowest (0.871, 0.871, 0.872, respectively) were slightly higher than that of FFR_CT_-2 cm alone (0.857), but without significnacne differences (*p* > 0.05 for all).

**Conclusions:**

FFR_CT_ measured at 2 cm distal to the lower border of the target lesion is the optimal measurement site for identifying lesion-specific ischemia in patients with CAD.

## Introduction

Coronary computed tomography angiography (CCTA) has been widely accepted as a reliable noninvasive assessment modality for excluding the presence of coronary artery significant obstructive disease (≥ 50% luminal narrowing) in low-to-intermediate-risk populations with a high negative predictive value [1–3]. However, its diagnostic specificity for assessing obstructive coronary artery disease (CAD) is still suboptimal. For example, in severely calcified plaques, luminal stenosis is often overestimated owing to calcium blooming. More importantly, CCTA cannot provide hemodynamic information to determine whether a particular stenotic lesion is associated with hemodynamically significant ischemia i.e., lesion-specific ischemia [4]. While exercise treadmill testing and stress echocardiography can assess overall ischemic burden, they are limited in locating lesion-specific ischemia on a per-vessel basis [5]. Therefore, accurate and prompt assessment of lesion-specific ischemia is critical in the management of stable CAD to improve its clinical outcomes and benefits as myocardial blood flow can be improved by medical therapy or revascularization procedures such as percutaneous coronary intervention (PCI) or coronary artery bypass graft surgery (CABG) [6].

Fractional flow reserve (FFR) is the gold standard for the assessment of lesion-specific ischemia to guide revascularization in stable CAD patients [7]. Clinically, FFR is measured along with invasive coronary angiography (ICA) by placing a pressure guidewire beyond a stenotic lesion and measuring the ratio of mean distal coronary pressure to mean aortic pressure ratio under conditions of adenosine infusion to the maximum hyperemia. At present, invasive FFR has become a cornerstone in determining lesion-specific ischemia and appropriate decision-making [8]. Whereas, invasive FFR requires additional expensive instruments, and clinical application of invasive FFR-informed treatment decision-making is relatively limited. Only 10% to 20% of revascularization procedures have incorporated invasive FFR results into the treatment decisions [9].

Recently, FFR can be noninvasively calculated from anatomical CCTA data based on computational fluid dynamics (CFD) [10]. This CT-based FFR (FFR_CT_) does not need additional imaging and vasodilator administration. FFR_CT_, the ratio of the maximum coronary blood flow through a stenotic artery to the blood flow assumed to be free of stenosis in that artery, has been validated to be useful in diagnosing and excluding lesion-specific ischemia [11–13]. Compared with invasive FFR, FFR_CT_ can provide FFR information almost at any site along the entire epicardial coronary artery tree [14]. Whereas the diagnostic accuracy of FFR_CT_ was previously determined by comparing a single measurement site corresponding to invasive FFR at a specific location within the coronary artery [11–13]. Inconsistent measurement sites between FFR_CT_ and invasive FFR can lead to inconclusive diagnostic results and confuse decision-making [15, 16]. Therefore, how to select an appropriate site along the coronary artery tree to measure FFR_CT_ is clinically relevant for the management of CAD. However, the optimal site to measure FFR_CT_ for a target lesion remains to be adequately determined.

In this single-center retrospective cohort study, CAD patients who had undergone both CCTA and invasive FFR were included. The diagnostic performances of FFR_CT_ measured at different sites at 1 cm, 2 cm, and 3 cm distal to the lower border of the target lesion of the artery (FFR_CT_-1 cm, FFR_CT_-2 cm, FFR_CT_-3 cm), and the lowest FFR_CT_ value at the distal vessel tip in which diameter larger than 1.5 mm (FFR_CT_-lowest) were assessed and compared with invasive FFR as the reference standard. The purpose of this study was to determine the optimal site to measure FFR_CT_ for a target lesion in detecting lesion-specific ischemia in CAD patients.

## Materials and methods

### Patients

**Fig. 1:**
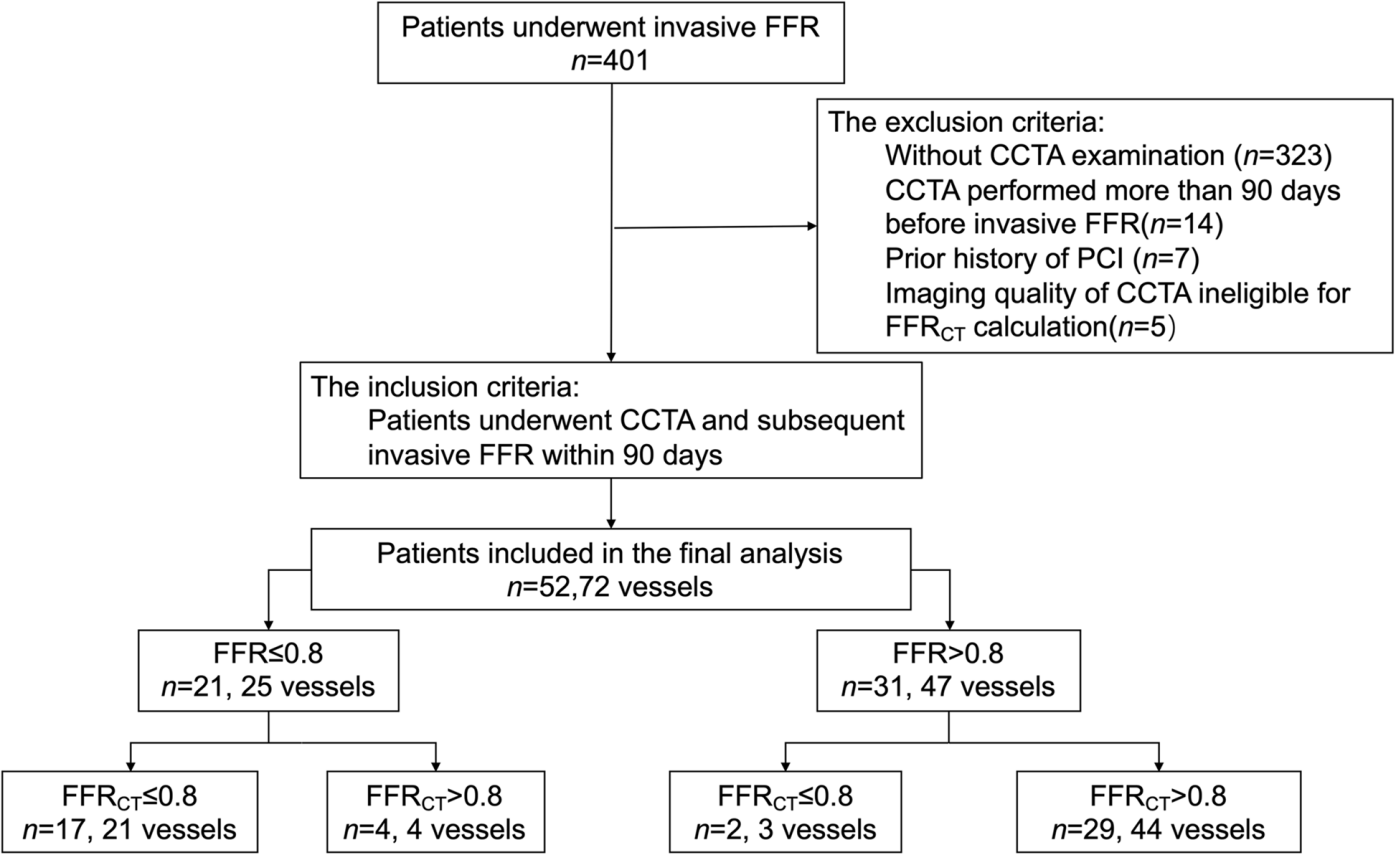
Flowchart shows patient enrollment and exclusion. CCTA, coronary computed tomography angiography; PCI, percutaneous coronary intervention; FFR, fractional flow reserve; FFR_CT_, computed tomography fractional flow reserve

This retrospective study was approved by the ethics review board of Sun Yat-Sen Memorial Hospital at Sun Yat-Sen University (SYSEC-KY-KS-2022–054; Guangzhou, China), and the need to obtain informed consent was waived. A total of 401 patients suspected of having CAD underwent invasive ICA and FFR between March 2017 and December 2021 were identified from the hospital database. Patients were included if they had undergone CCTA and subsequent invasive FFR within 90 days. The exclusion criteria were as follows: without CCTA examination (*n* = 323), CCTA performed more than 90 days before invasive FFR (*n* = 14), prior history of PCI (*n* = 7), or imaging quality of CCTA ineligible for FFR_CT_ calculation (*n* = 5). Finally, a total of 52 patients with 72 coronary vessels were included for analysis. The flowchart of the patient enrollment pathway is shown in Fig. 1. The demographics and baseline clinical characteristics of 52 patients are listed in Table 1.

**Table 1:** Demographics and baseline clinical characteristics of 52 patients with CAD

Characteristics	All Patients	Lesion-specific ischemia		*p* value
		Present	Absent	
No. of patients	52	21 (40.4)	31 (59.6)	
Age (years) ^a^	66.7 ± 7.9	64.3 ± 8.6	68.5 ± 7.2	0.064
Gender				0.001
Male	25 (48.1)	16 (64.0)	9 (29.0)	
Female	27 (51.9)	5 (36.0)	22 (71.0)	
BMI(kg/m ^2^) ^a^	24.8 ± 3.5	25.5 ± 3.7	24.3 ± 3.3	0.236
EF (%) ^a^	69.4 ± 4.4	69.6 ± 4.6	69.3 ± 4.4	0.816
Risk factors				
Hypertension	31/52(59.6)	12/21 (57.1)	19/31 (61.3)	0.781
Diabetes mellitus	13/52(25.0)	5/21 (23.8)	8/31 (26.8)	1.000
Hyperlipidemia	22/52(42.3)	11/21 (52.4)	11/31 (35.5)	0.263
Smoking	13/52(25.0)	6/21 (28.6)	7/31 (22.6)	0.747
NYHA Classification				0.523
I	39/52(75.0)	17 (81.0)	22 (71.0)	
II	13/52(25.0)	4 (19.0)	9 (29.0)	
CAD in target vessels				
No. of vessels	72	25 (34.7)	47 (65.3)	
LAD	44 (61.1)	20 (45.5)	24 (54.5)	
LCX	12 (16.7)	3 (25.0)	9 (75.0)	
RCA	16 (22.2)	2 (12.5)	14 (87.5)	
CCTA (DS ≥ 50%)	63 (87.5)	25 (39.7)	38 (60.3)	
Interval between CCTA and invasive FFR (day) †	17.5(4.5–72)	11(5–39.5)	37(2–76)	0.815

Unless otherwise specified, data are numbers of patients, with percentages in parentheses. *BMI* body mass index, *EF* ejection fraction, *NYHA* New York Heart Association. CAD, coronary artery diseases, *LAD* left anterior descending artery, *LCX* left circumflex artery, *RCA* right coronary artery, *CCTA* coronary CT angiography, *DS* diameter stenosis

^a^Data are means ± standard deviations. † Data are medians, with interquartile ranges in parentheses

### ICA and invasive FFR measurement

All patients underwent conventional ICA and invasive FFR using standard techniques [17]. Each of the three main coronary vessels (anterior descending branch, circumflex branch and right coronary) was included in the analysis. All vessel segments were evaluated by two cardiologists (Y.L., with 20 years of experience of coronary intervention, and H.Z., with 15 years of experience of coronary intervention). Vessels with 30%-90% diameter stenosis as determined by ICA were referred to invasive FFR evaluation. For each vessel, if only one lesion having 30%-90% diameter stenosis was present, this lesion was selected as the target lesion according to the DeFACTO study [12]. If serial lesions were present, the lesion with 30%-90% diameter stenosis most distal to the vessel end was chosen as the target lesion according to the study by Nozaki et al. [18]. Lesion < 30% stenosis was not chosen as target lesion. For invasive FFR, a pressure-monitoring guidewire (St Jude Medical, Minneapolis, Minn) was advanced 2–3 cm distal to the target lesion after administration of nitroglycerin [19]. Hyperemia was attained by intravenous administration of adenosine 5'-triphosphate at a dosage of 160 ug/kg/min. The lesion was considered lesion-specific ischemia when the measured FFR was ≤ 0.8.

### CCTA protocol

CCTA was performed on a 64-slice CT scanner (SOMATOM Sensation 64; Siemens Healthineers, Forchheim, Germany or Discovery CT750HD; GE Healthcare, Pewaukee, WI, USA) with a retrospective ECG-gated technique or a third-generation 192-slice dual-source CT scanner (SOMATOM Force, Siemens Healthineers, Forchheim, Germany) with a prospective ECG-gated technique. The detailed CCTA protocols are listed in Table 2. Patient preparation and CT scanning were performed according to the Society of Cardiovascular Computed Tomography (SCCT) guidelines [20]. Patients with a heart rate > 70 beats/min were given oral beta-blockers (metoprolol tartrate tablets, 25–75 mg, Astrazeneca Pharmaceuticals China Co., LTD) 2 h before the CT scanning. In the absence of contraindications (hypotension, severe anemia, current use of nitrate medications, known nitroglycerin allergy), patients were given a 0.5 mg nitroglycerin tablet (Peking Yimin Pharmaceutical Co., Ltd) sublingually 2 min before scanning. CCTA images were obtained after intravenous injection of iodinated nonionic contrast agent (Iohexol; 350 mg/dl iodine, GE Healthcare, Cork, Ireland) at a dose of 1.0 ml/kg body weight with an infusion rate of 5 ml/s, followed by the injection of 40 ml saline at the same flow rate using a dual-head injector (Medrad stellant CT injector system; Medrad, Bayer Medical Care Inc, Indianola, PA, USA). Automatic bolus-tracking technology was used. The region of interest (ROI) was set at the ascending aorta of the aortopulmonary fenestration plane, and the triggering threshold value was 100 Hounsfield units. The scan range included the whole heart from the superior border of the aortic arch to the diaphragmatic surface of the heart. The CT technologists (H.H., with 20 years of experience in ECG-gated cardiac CT scanning) determined the optimal stationary cardiac phase images with minimum motion-free and transferred them to an offline workstation for further analyses.

**Table 2:** Acquisition parameters of CCTA protocol

CT Scanners	Collimation	Rotation time	kVp	mAs	Slice thickness	Slice interval	Matrix	FOV
SOMATOM Force	96 × 0.6 mm	250 ms	70–100	320	0.6 mm	0.5 mm	512	211 mm
SOMATOM Sensation	64 × 0.6 mm	330 ms	120	900	0.6 mm	0.5 mm	512	211 mm
Discovery 750 HD	64 × 0.625 mm	330 ms	120	228	0.625 mm	0.625 mm	512	194 mm

*FOV* field of view

### Morphologic analysis of CCTA

The CCTA images were analyzed by two radiologists with more than five years of experience (M.C., with 8 years of experience in cardiac imaging and G.S., with 7 years of experience in cardiac imaging) who were blinded to the ICA and invasive FFR results except for the location of the target lesion. The location of target lesion on CCTA was marked by the radiologist and the cardiologist together. The percentage diameter stenosis of the target lesion was measured using an offline quantitative coronary CT angiography software (Syngo·Via, Siemens Healthineers, Forchheim, Germany). Curved multiplanar reconstructions, maximum intensity projection (MIP), and volume rendering technique (VRT) were used to generate diagnostic images for interpretation. Lumen stenosis ≥ 50% was defined as significant obstruction according to the SCCT guidelines [21].

### FFR_CT_ analysis

FFR_CT_ analysis was performed using a dedicated software (RuiXin-FFR_CT_, Raysight Inc., Shenzhen, Guangdong Province, China) which based on CFD to calculate FFR_CT_ using CCTA images. First, the initial segmentation model of the entire coronary artery was established, upon which the center line and contour of each coronary artery were obtained by the region growth algorithm. The contour was connected and smoothen to obtain a 3D model of the whole coronary artery. Then, the morphologic data of the heart were obtained according to CT images; Combined with the statistical prediction model of the basic characteristics of the patient (such as allometric growth law), the unique physiological indexes of the patient were obtained, such as the coronary artery pressure, coronary artery flow in the state of maximum congestion, microcirculation resistance, etc. Finally, an unstructured mesh was generated on the 3D model, and the blood was assumed to be a Newtonian liquid. The incompressible Navier Stokes equation was solved using the finite element algorithm to obtain the pressure and velocity of each grid point in the whole 3D coronary artery model, and then the FFR_CT_ value was obtained.

**Fig. 2:**
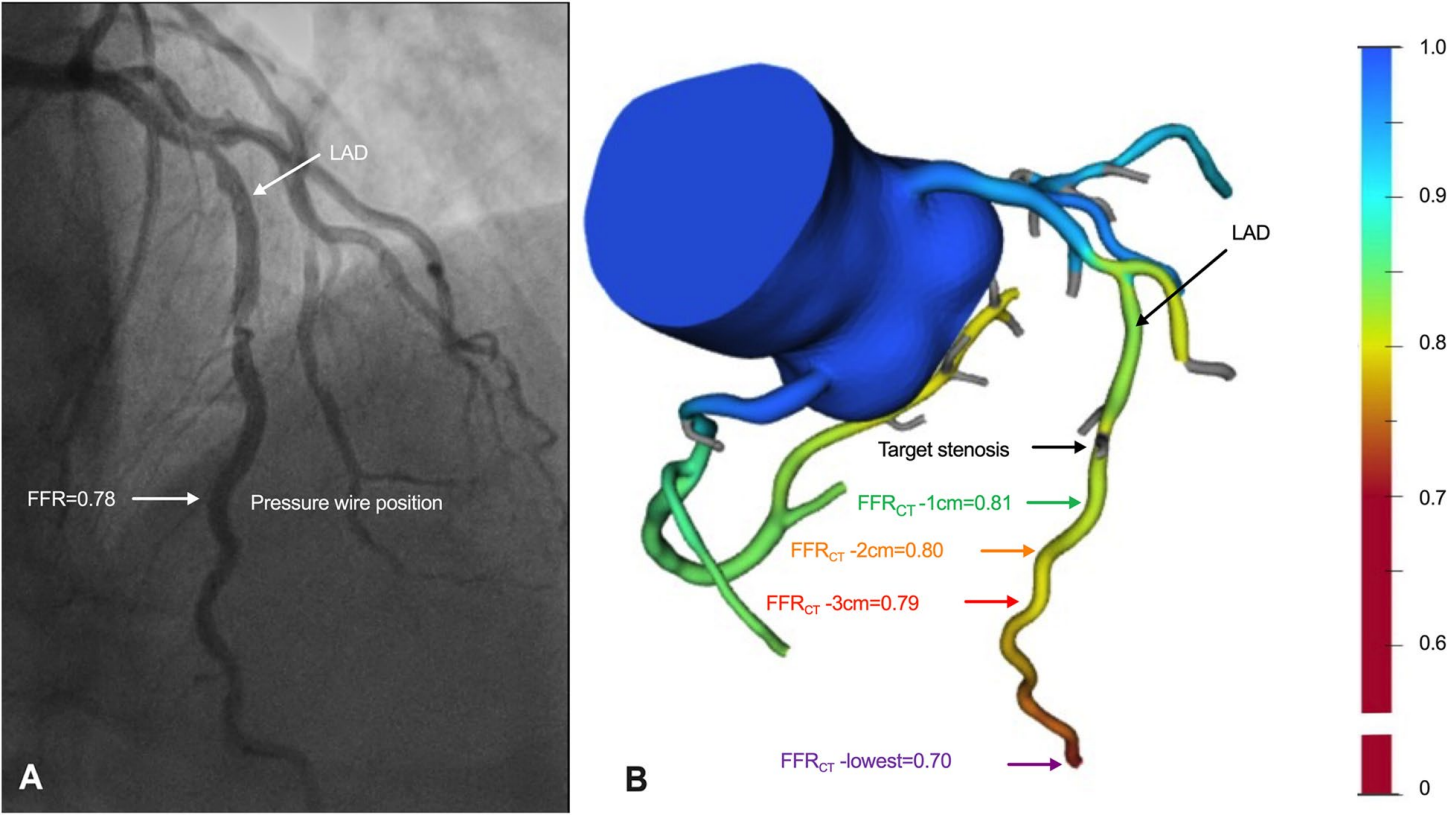
Representative images for invasive FFR and FFR_CT_ measurements. **A** ICA image showing the tip of pressure-wire position in left anterior descending artery (LAD) during invasive FFR and the measured FFR was 0.78. **B** The corresponding pseudo-colorized FFR_CT_ image show the site of target lesion and the measured FFR_CT_ at 1, 2, 3 cm distal to the target lesion (FFR_CT_-1 cm, FFR_CT_-2 cm FFR_CT_-3 cm) and the lowest FFR_CT_ (FFR_CT_-lowest). LAD, left anterior descending artery; FFR, fractional flow reserve; FFR_CT_, CT fractional flow reserve; FFR_CT_-1 cm, FFR_CT_ measured at 1 cm distal to the lower border of the target lesion; FFR_CT_-2 cm, FFR_CT_ measured at 2 cm distal to the lower border of the target lesion; FFR_CT_-3 cm, FFR_CT_ measured at 3 cm distal to the lower border of the target lesion; FFR_CT_-lowest, lowest FFR_CT_ value in the distal vessel tip

For each coronary artery, two radiologists (Z.Y., with fifteen years of experience in cardiac imaging; T.Y., with ten years of experience in cardiac imaging) who did not know the results of invasive FFR except for the location of target lesion independently measured FFR_CT_ at four sites as follows (Fig. 2), at 1 cm, 2 cm, 3 cm distal to the lower border of the target lesion of the artery (FFR_CT_-1 cm, FFR_CT_-2 cm, FFR_CT_-3 cm), and the lowest FFR_CT_ value in the distal vessel tip (FFR_CT_-lowest, coronary modeling with FFR_CT_ data was limited to coronary vessels with a minimum luminal diameter of ≥ 1.5 mm), the position of FFR_CT_-1 cm, FFR_CT_-2 cm, FFR_CT_-3 cm were measured on a reconstructed curved planar image. FFR_CT_ value of ≤ 0.80 was considered to be lesion-specific ischemia.

### Statistical analysis

The normality of quantitative data was assessed using the Shapiro–Wilk test. Descriptive statistics were presented as mean ± standard deviation (SD) for normally distributed variables. Non-normally distributed variables were expressed as the median and interquartile range (IQR), and categorical variables were expressed as numbers of cases (and percentages). Intraclass correlation coefficients (ICC) with a 95% confidence interval (CI) were used to assess the interobserver agreement in FFR_CT_-1 cm, FFR_CT_-2 cm, FFR_CT_-3 cm and FFR_CT_-lowest measurements. Data from two radiologists were averaged for analysis. Pearson's correlation analysis was used to evaluate the relationship between invasive FFR and FFR_CT_ values. Correlation coefficients derived from Chi-suqare test were used to assess the correlation between invasive FFR and the cominbaiton of FFR_CT_ measred at four sites. Bland–Altman plots were used to visualize the differences of invasive FFR and FFR_CT_ values. With invasive FFR as the reference standard, the accuracy, sensitivity, specificity, positive predictive value (PPV), and negative predictive value (NPV) of FFR_CT_, the combination of FFR_CT_ measured at two or three sites, CCTA (significant obstruction stenosis, diameter stenosis ≥ 50%), and CCTA combined with each of FFR_CT_ measured at four sites in detecting lesion-specific ischemia were calculated. The performances of CCTA, FFR_CT_ measured at the four different sites and their combination in diagnosing lesion-specific ischemia were evaluated by receiver-operating characteristic (ROC) curves. The areas under the curve (AUCs) were compared using the DeLong method [22]. The sensitivities and specificities of FFR_CT_ and CCTA were compared by Chi-square, Fisher exact test, or McNemar test. Statistical analysis was performed using SPSS Statistics version 26 (IBM corporation, Armonk, NY, USA) or R 3.3.3 (R Foundation for Statistical Computing, Vienna, Austria) software. A *p* value < 0.05 was considered the significant threshold.

## Results

### Invasive FFR and FFR_CT_

A total of 52 patients were included. Twenty-five vessels in 21 patients had lesion-specific ischemia as detected by invasive FFR. Forty-seven vessels in 31 patients had no lesion-specific ischemia as detected by invasive FFR. More male patients had lesion-specific ischemia (64.0% vs. 29.0%, *p* < 0.05) than female patients (Table 1). No significant differences were found in age, ejection fraction, New York Heart Association (NYHA) class, and body mass index (BMI) between patients with and without the lesion-specific ischemia (*p* > 0.05, Table 1). Invasive FFR and FFR_CT_ of 72 vessels in 52 patients with CAD are listed in Table 3. FFR_CT_-1 cm, FFR_CT_-2 cm, FFR_CT_-3 cm, and FFR_CT_-lowest in vessels with lesion-specific inshcemia were lower than those in vessels without lesion-specific inshcemia (*p* < 0.05 for all).

**Table 3:** Invasive FFR and FFR_CT_ of 72 vessels in 52 patients

Characteristics	All vessels	Lesion-specific ischemia		*p* value
		Present	Absent	
Invasive FFR	0.86 (0.77–0.93)	0.75 (0.67–0.78)	0.91 (0.86–0.95)	< 0.05
FFR_CT_-1 cm	0.87 (0.80–0.92)	0.80 (0.77–0.84)	0.90 (0.86–0.94)	< 0.05
FFR_CT_-2 cm	0.85 (0.79–0.91)	0.78 (0.82–0.80)	0.88 (0.84–0.93)	< 0.05
FFR_CT_-3 cm	0.85 (0.78–0.90)	0.77 (0.70–0.79)	0.88 (0.83–0.92)	< 0.05
FFR_CT_-lowest	0.80 (0.75–0.89)	0.73 (0.67–0.79)	0.86 (0.80–0.91)	< 0.05

Data are medians, with interquartile ranges in parentheses. *FFR*_*CT*_ CT fractional flow reserve, *FFR* fractional flow reserve

### Interobserver agreement

The ICCs in FFR_CT_ measurements between the two observers were 0.97 (95% CI, 0.96 to 0.98) for FFR_CT_-1 cm, 0.98 (95% CI, 0.97 to 0.99) for FFR_CT_-2 cm, 0.99 (95% CI, 0.98 to 0.99) for FFR_CT_-3 cm, and 1.00 (95% CI, 0.99 to 1.00) for FFR_CT_-lowest.

### Correlation between FFR_CT_ and invasive FFR

**Fig. 3:**
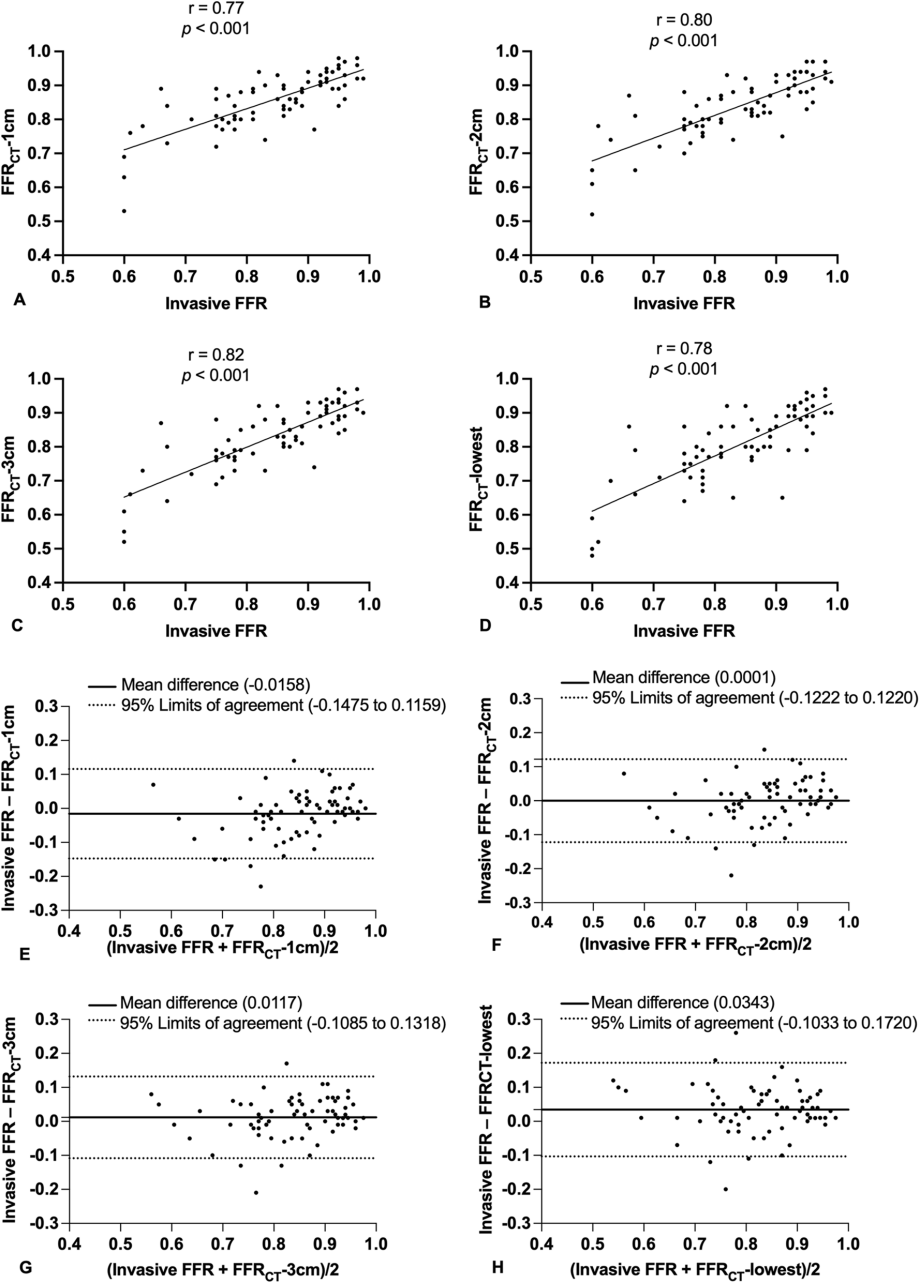
Scatter plots show a moderate correlation between invasive FFR and FFR_CT_-1 cm (**A**) and between invasive FFR and FFR_CT_-lowest (**B**), a good correlation between invasive FFR and FFR_CT_-2 cm (**C**) and between invasive FFR and FFR_CT_-3 cm (**D**). Bland–Altman plots show a very mild difference between invasive FFR and FFR_CT_-1 cm (**E**), invasive FFR and FFR_CT_-2 cm (**F**), invasive FFR and FFR_CT_-3 cm (**G**), invasive FFR and FFR_CT_-lowest (**H**) on a per-vessel bias. FFR, fractional flow reserve; FFR_CT_, CT fractional flow reserve; FFR_CT_-1 cm, FFR_CT_ measured at 1 cm distal to the lower border of the target lesion; FFR_CT_-2 cm, FFR_CT_ measured at 2 cm distal to the lower border of the target lesion; FFR_CT_-3 cm, FFR_CT_ measured at 3 cm distal to the lower border of the target lesion; FFR_CT_-lowest, lowest FFR_CT_ value in the distal vessel tip

Correlation analysis showed that there was a good correlation between invasive FFR and FFR_CT_-2 cm(*r* = 0.80 95% CI, 0.70 to 0.87,* p* < 0.001) and between invasive FFR and FFR_CT_-3 cm (*r* = 0.82, 95% CI, 0.72 to 0.88, *p* < 0.001), there was a moderate correlation between invasive FFR and FFR_CT_-1 cm (*r* = 0.77, 95% CI, 0.65 to 0.85,* p* < 0.001), and between invasive FFR and FFR_CT_-lowest (*r* = 0.78, 95% CI, 0.67 to 0.86, *p* < 0.001) (Fig. 3A-D). Bland–Altman plots showed a mild difference between invasive FFR and FFR_CT_ on a per-vessel bias (invasive FFR vs. FFR_CT_-1 cm, mean difference -0.0158, 95% limits of agreement: -0.1475 to 0.1159; invasive FFR vs. FFR_CT_-2 cm, mean difference 0.0001, 95% limits of agreement: -0.1222 to 0.1220; invasive FFR vs. FFR_CT_-3 cm, mean difference 0.0117, 95% limits of agreement: -0.1085 to 0.1318; and invasive FFR vs. FFR_CT_-lowest, mean difference 0.0343, 95% limits of agreement: -0.1033 to 0.1720) (Fig. 3E-H). Correlation analyses showed that FFR_CT_-1 cm + FFR_CT_-2 cm, FFR_CT_-2 cm + FFR_CT_-3 cm, FFR_CT_-3 cm + FFR_CT_-lowest,FFR_CT_-1 cm + FFR_CT_-2 cm + FFR_CT_-3 cm, and FFR_CT_-2 cm + FFR_CT_-3 cm + FFR_CT_-lowest were correatled with invasive FFR (*r* = 0.722; 0.722; 0.701; 0.722; and 0.722, respectively; *p* < 0.001 for all).

### Optimal measurement site of FFR_CT_

**Fig. 4:**
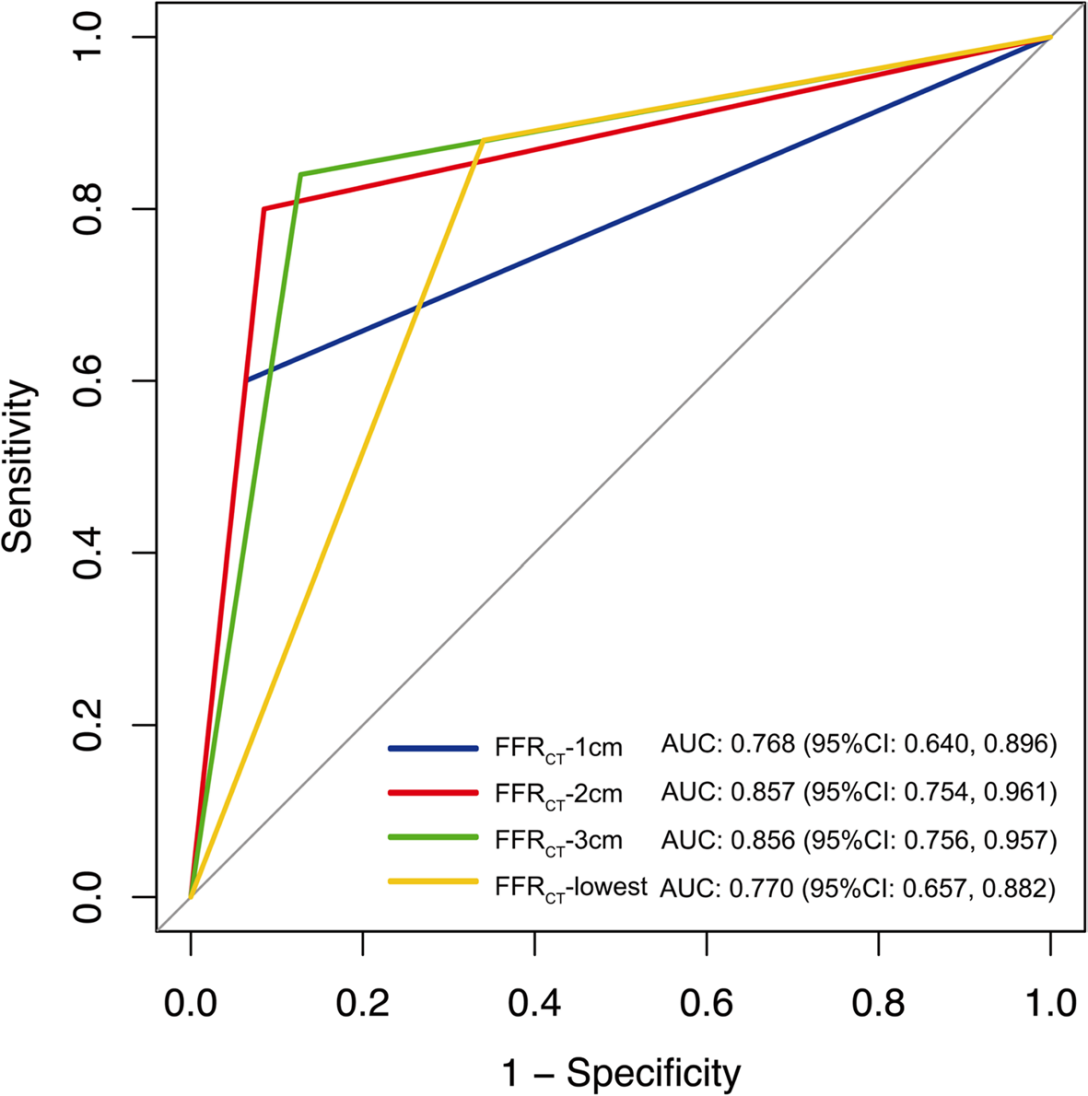
Receiver operating characteristic curves of the FFR_CT_-1 cm, FFR_CT_-2 cm, FFR_CT_-3 cm, FFR_CT_-lowest per-vessel in identifying lesion ischemia with invasive FFR as the reference standard based on per-vessel analysis. The AUCs of FFR_CT_-2 cm and FFR_CT_-3 cm in detecting lesion-specific ischemia were significantly higher than FFR_CT_-1 cm and the FFR_CT_-lowest (*p* < 0.05 in each case). AUC, area under the curve

ROC analyses of FFR_CT_-1 cm, FFR_CT_-2 cm, FFR_CT_-3 cm, FFR_CT_-lowest per-vessel in identifying lesion-specific ischemia are shown in Table 4. The diagnostic sensitivity and NPV increased gradually with increasing of the distance from target lesion among the different measurement sites. FFR_CT_-lowest had lower accuracy and PPV than FFR_CT_-2 cm, while had higher sensitivity than FFR_CT_-2 cm (accuracy, *p* = 0.035; sensitivity,* p* = 0.002, and PPV, *p* = 0.037, respectively). FFR_CT_-lowest ≤ 0.8 was found in 51.3% vessels (38/72) while FFR_CT_-2 cm ≤ 0.8 was found in 33.3% (24/72) vessels. 18% vessels with FFR_CT_-lowest ≤ 0.8 were reclassified as negative according to their FFR_CT_-2 cm values. There was no statistical significance in accuracy, sensitivity and NPV between FFR_CT_-1 cm and FFR_CT_-2 cm (accuracy, *p* = 0.123; sensitivity, *p* = 0.977, and NPV, *p* = 0.249 respectively). The AUCs of FFR_CT_-1 cm, FFR_CT_-2 cm, FFR_CT_-3 cm, FFR_CT_-lowest per-vessel in identifying lesion-specific ischemia using invasive FFR as reference standard were 0.768 (95% CI, 0.640 to 0.896), 0.857 (95% CI, 0.754 to 0.961), 0.856 (95% CI, 0.756 to 0.957) and 0.770 (95% CI, 0.657 to 0.882), respectively **(**Fig. 4), with the AUC of FFR_CT_-2 cm being the greatest. The AUCs of FFR_CT_-1 cm and FFR_CT_-lowest were significantly lower than that of FFR_CT_-2 cm (0.770 vs. 0.857 for FFR_CT_-lowest vs. FFR_CT_-2 cm, *p* < 0.05; 0.768 vs. 0.857 for FFR_CT_-1 cm vs. FFR_CT_-2 cm,* p* < 0.05). The AUCs showed no statistical significance between FFR_CT_-2 cm and FFR_CT_-3 cm (*p* = 0.29*5*).

**Table 4:** ROC analysis of CCTA, FFR_CT_-1 cm, FFR_CT_-2 cm, FFR_CT_-3 cm, FFR_CT_-lowest and their combination in identifying lesion-specific ischemia on per-vessel basis

Per-vessel	Accuracy (%)	Sensitivity (%)	Specificity (%)	PPV (%)	NPV (%)	AUC	*P value*
CCTA (DS ≥ 50%)	47.2 (34/72) [36.1, 58.6]	100.0(25/25) [83.4, 100.0]	19.1(9/47) [9.6, 33.7]	39.7(25/63) [27.8, 52.8]	100.0 (9/9) [62.9, 100.0]	0.578 [0.443, 0.713]	0.277
FFR_CT_-1 cm	81.9(59/72) [79.5, 89.1]	60.0(15/25) [38.9, 78.2]	93.6(44/47) [81.4, 98.3]	83.3(15/18) [57.7, 95.6]	81.5(44/54) [68.1, 90.3]	0.768 [0.640, 0.896]	0.0001
FFR_CT_-2 cm	87.5(63/72) [77.9 93.3]	80.0(20/25) [58.7, 92.4]	91.5(43/47) [78.7, 97.2]	83,3(20/24) [61.8, 94.5]	89.6(43/48) [76.6, 96.1]	0.857 [0.754, 0.961]	0.0001
FFR_CT_-3 cm	86.1(62/72) [76.3 92.3]	84.0(21/25) [63.1, 94.7]	87.2(41/47) [71.6, 93.5]	77.8(21/27) [54.8,88.6]	88.9(41/45) [77.9, 88.6]	0.856 [0.756, 0.957]	0.0001
FFR_CT_-lowest	73.6(53/72) [62.4, 82.4]	88.0(22/25) [67.7, 96.8]	66.0(31/47) [50.6, 78,7]	58.0(22/38) [40.9, 73.3]	91.2(31/34) [75.2, 97.7]	0.770 [0.657, 0.882]	0.0001
FFR_CT_-1 cm + FFR_CT_-2 cm	87.5(63/72) [77.9 93.3]	80.0(20/25) [58.7, 92.4]	91.5(43/47) [78.7, 97.2]	83,3(20/24) [61.8, 94.5]	89.6(43/48) [76.6, 96.1]	0.857 [0.754, 0.961]	0.0001
FFR_CT_-2 cm + FFR_CT_-3 cm	87.5(63/72) [77.9 93.3]	80.0(20/25) [58.7, 92.4]	91.5(43/47) [78.7, 97.2]	83,3(20/24) [61.8, 94.5]	89.6(43/48) [76.6, 96.1]	0.871 [0.774, 0.969]	0.0001
FFR_CT_-3 cm + FFR_CT_-lowest	86.1(62/72) [76.3 92.3]	84.0(21/25) [63.1, 94.7]	87.2(41/47) [71.6, 93.5]	77.8(21/27) [54.8,88.6]	88.9(41/45) [77.9, 88.6]	0.857 [0.756, 0.957]	0.0001
FFR_CT_-1 cm + FFR_CT_-2 cm + FFR_CT_-3 cm	87.5(63/72) [77.9 93.3]	80.0(20/25) [58.7, 92.4]	91.5(43/47) [78.7, 97.2]	83,3(20/24) [61.8, 94.5]	89.6(43/48) [76.6, 96.1]	0.871 [0.774, 0.969]	0.0001
FFR_CT_-2 cm + FFR_CT_-3 cm + FFR_CT_- lowest	87.5(63/72) [77.9 93.3]	80.0(20/25) [58.7, 92.4]	91.5(43/47) [78.7, 97.2]	83,3(20/24) [61.8, 94.5]	89.6(43/48) [76.6, 96.1]	0.872 [0.774, 0.970]	0.0001
CCTA + FFR_CT_-1 cm	81.9(59/72) [79.5, 89.1]	60.0(15/25) [38.9, 78.2]	93.6(44/47) [81.4, 98.3]	83.3(15/18) [57.7, 95.6]	81.5(44/54) [68.1, 90.3]	0.785 [0.664, 0.905]	0.0001
CCTA + FFR_CT_-2 cm	87.5(63/72) [77.9 93.3]	80.0(20/25) [58.7, 92.4]	91.5(43/47) [78.7, 97.2]	83,3(20/24) [61.8, 94.5]	89.6(43/48) [76.6, 96.1]	0.868 [0.770, 0.967]	0.0001
CCTA + FFR_CT_-3 cm	86.1(62/72) [76.3 92.3]	84.0(21/25) [63.1, 94.7]	87.2(41/47) [71.6, 93.5]	77.8(21/27) [54.8,88.6]	88.9(41/45) [77.9, 88.6]	0.863 [0.765, 0.961]	0.0001
CCTA + FFR_CT_-lowest	73.6(53/72) [62.4, 82.4]	88.0(22/25) [67.7, 96.8]	66.0(31/47) [50.6, 78,7]	58.0(22/38) [40.9, 73.3]	91.2(31/34) [75.2, 97.7]	0.798 [0.695, 0.902]	0.0001

*PPV* positive predictive value, *NPV* negative predictive value, *CCTA* coronary computed tomography angiography, *DS* diameter stenosis, *FFR*_*CT*_*-1 cm* FFR_CT_ measured at 1 cm distal to the lower border of the target lesion, *FFR*_*CT*_*-2 cm* FFR_CT_ measured at 2 cm distal to the lower border of the target lesion, *FFR*_*CT*_*-3 cm* FFR_CT_ measured at 3 cm distal to the lower border of the target lesion, *FFR*_*CT*_*-lowest* lowest FFR_CT_ value in the distal vessel tip. Data in parentheses are number of vessels. Data in brackets are 95% confidence interval

The AUCs were similar between FFR_CT_-1 cm + FFR_CT_-2 cm (0.857 [95% CI, 0.754 to 0.961]), FFR_CT_-3 cm + FFR_CT_-lowest (0.857 [95% CI, 0.756 to 0.957]) and FFR_CT_-2 cm alone (0.857 [95% CI, 0.756 to 0.957]) ( *p* > 0.05 for all). The AUCs of FFR_CT_-2 cm + FFR_CT_-3 cm (0.871 [95% CI, 0.774 to 0.969]), FFR_CT_-1 cm + FFR_CT_-2 cm + FFR_CT_-3 cm (0.871 [95% CI, 0.774 to 0.969]), and FFR_CT_-and 2 cm + FFR_CT_-3 cm + FFR_CT_-lowest (0.872 [95% CI, 0.774 to 0.970]) were slightly higher than that of FFR_CT_-2 cm alone (0.857 [95% CI, 0.756 to 0.957]), but without significnacne differences (*p* > 0.05 for all).

### Additive value of FFR_CT_

The AUCs of CCTA (DS ≥ 50%) in identifying lesion-specific ischemia using invasive FFR as reference standard were 0.576 (95% CI, 0.443 to 0.713) (Table 4). The AUCs of FFR_CT_ measured at all the four different sites were significantly higher than CCTA DS (all* p* < 0.05). CCTA combined with any FFR_CT_ measured at 4 different sites had a higher AUC than CCTA alone (CCTA + FFR_CT_-1 cm, 0.785 [95% CI, 0.664 to 0.905]; CCTA + FFR_CT_-2 cm, 0.868 [95% CI, 0.770 to 0.967]; CCTA + FFR_CT_-3 cm, 0.863 [95% CI, 0.765 to 0.961]; and CCTA + FFR_CT_-lowest, 0.798 [95% CI, 0.695 to 0.902]; *p* < 0.01 for all).

## Discussion

In this study, FFR_CT_ measured at four different sites along the coronary arteries was used to identify lesion-specific ischemia using invasive FFR as the reference standard. Our results showed that FFR_CT_-2 cm had the highest accuracy (87.5%) and AUC (0.857) in identifying lesion-specific ischemia and FFR_CT_-lowest has the highest sensitivity and NPV than FFR_CT_-1 cm, FFR_CT_-2 cm, FFR_CT_-3 cm. CCTA (DS ≥ 50%) combined with either FFR_CT_-1 cm, FFR_CT_-2 cm, FFR_CT_-3 cm, or FFR_CT_-lowest showed higher AUC than CCTA alone in detecting lesion-specific ischemia.

Commonly, invasive FFR measurement is performed at one position selected 2-to-3 cm distal to the target lesion along coronary artery tree during ICA [19]. Unlike invasive FFR, FFR_CT_ values can be obtained along the entire coronary arterial tree. Inappropriate FFR_CT_ measurement can mislead the clinical decision. However, there is no consensus about the best location to measure FFR_CT_ in clinical practice. Previously, Cami et al. [16] used a CFD-based algorithm to measure FFR_CT_ value 10.5 mm (IQR 7.3–14.8 mm) distal to the stenosis in 26 patients with proximal LAD stenosis and found that it was a reliable location for measuring FFR_CT_ using invasive FFR measured 2-3 cm distal to the stenosis as reference standard. An expert panel [23] based on the finding of Cami et al. [16] advised to use the minimum FFR_CT_ values measured 1 to 2 cm distal to the stenosis as the result to judge the coronary lesion-specific ischemia. However, the measurement site proposed by Cami et al. [16] was defined as the distance from the distal end of the target stenosis to the place where FFR_CT_ declined to a lower plateau. In a similar study by Omori et al. [15], the diagnostic performance of FFR_CT_ measured at 1 to 2 cm distal to the stenosis was also found to be higher than that of FFR_CT_-lowest (0.86 vs. 0.80, *p* = 0.002) in identifying lesion-specific ischemia using invasive FFR as reference standard. In addition, Nozaki et al. [18] used a CFD-based algorithm and found that the AUC of FFR_CT_-2 cm was higher than that of FFR_CT_-lowest (0.80 vs. 0.68, *p* = 0.002) in identifying lesion-specific ischemia and was comparable with that of FFR_CT_-1 cm (0.80 vs. 0.79, *p* = 0.73). In a recent study by Chen et al. [24] where a machine learning-based algorithm was applied, the AUC of FFR_CT_-2 cm was found to be comparable with that of FFR_CT_-1 cm (0.91 vs. 0.91, *p* = 0.663) and was higher than that of FFR_CT_-3 cm (0.91 vs. 0.88, *p* = 0.002) and FFR_CT_-4 cm (0.91 vs. 0.88, *p* = 0.008) in identifying lesion-specific ischemia using invasive FFR as reference standard. Based on these results, FFR_CT_ measured at 1-to-2 cm distal to the stenosis is better than FFR_CT_-lowest in identifying lesion-specific ischemia in patients with CAD.

In our study, invasive FFR was also used as the reference standard. Our results showed that there was a good correlation (*r* = 0.80,* p* < 0.001) and a very mild difference (mean difference 0.0001, 95% limits of agreement: -0.1222 to 0.1220) between invasive FFR and FFR_CT_-2 cm. Further ROC analysis showed that the diagnostic performance of FFR_CT_ measured at 2 cm distal to the target lesion (FFR_CT_-2 cm) was higher than that measured at 1 cm distal to the target lesion (FFR_CT_-1 cm) and that measured at the vessel terminus (FFR_CT_-lowest) (AUC: 0.857, 0.768, 0.770, respectively), and was comparable with FFR_CT_-3 cm (AUC: 0.856). These findings suggest that FFR_CT_ measured at 2 cm distal to the target lesion had the highest performance in identifying lesion-specific ischemia. Overall, our results are consistent with the findings of previous study [15, 16, 18, 24]. In our study, FFR_CT_ was measured at four different sites along the same artery and our results showed that FFR_CT_ measured at 2 cm distal to the target lesion are the optimal site for FFR_CT_ measurement, which is in line with previous studies [15, 16, 18, 24]. It should be noted that the measurement site of the invasive FFR and the definition of the target lesion were not totally consistent between our study and previous studies [15, 16, 18, 24]. Indeed, the invasive FFR was measured 2 to 3 cm distal to the target lesion in our study which was based on the recommendation as previously described [19],which is as same as the study by Cami et al. [16]. Nonetheless, Omori et al. [15] used invasive FFR measured 2–4 cm distal to the target lesion as the reference standard, Nozaki et al. [18] used invasive FFR measured distal to the stenosis as far as possible as the reference standard. Chen et al. [24] used invasive FFR which was measured at a minimum of 2 cm distal to the stenosis in vessel segments ≥ 2 mm as the reference standard. As regards the target lesion, the definition of the target lesion of a serial lesion in the study by Nozaki et al. [18] was similar to our study, i.e., the most distal lesion in the vessel with 30%-90% diameter stenosis selected as the target lesion. However, Omori et al. [15] and Chen et al. [24] selected the most severe stenosis in a serial lesion as the target lesion. These differences likely result in the mild discrepancy in the diagnostic performances of FFR_CT_ measured at 1 to 2 cm distal to the target lesion. It is known that there is a gradual decrease in FFR_CT_ from proximal to distal along the vessel even without focal stenosis [16]. The lowest value of FFR_CT_ measured at 1 to 2 cm distal to the target lesion probably is not significantly different from FFR_CT_ measured 2 cm distal to the lower border of the target lesion. Thus, it is reasonable that 2 cm distal to the target lesion could be used as the exact measurement site for FFR_CT_. Our results indicate that FFR_CT_-2 cm is the optimal for identifying lesion-specific ischemia. This finding might impact the clinical decision-making and patient outcomes. For example, if a patient has a lesion with 30–90% vessel diameter stenosis while no lesion-specific ischemia as determined by FFR_CT_-2 cm, this patient could avoid invasive FFR and unnecessary interventional revascularization.

Due to the presence of a gradual decrease in FFR_CT_ from proximal to distal along the vessel even without focal stenosis [16], FFR_CT_ measures at different sites along the same coronary arterial might have different clinical indication. Our study showed that FFR_CT_-1 cm has the highest specificity (93.6%) in diagnosing lesion-specific ischemia compared with FFR_CT_-2 cm, FFR_CT_-3 cm and FFR_CT_-lowest, but had the lowest sensitivity (60%); FFR_CT_-lowest has the highest sensitivity (88%) and NPV (91.2%) in identifying lesion-specific ischemia but had the lowest specificity (66%) and PPV (58%). 18% vessels positive for FFR_CT_-lowest were reclassified as negative when determined by FFR_CT_-2 cm. These results indicate that FFR_CT_-1 cm could underestimate the severity of the lesion and the FFR_CT_-lowest could overestimate the severity of the lesion. Similarly, Kueh et al. [25] also found that FFR_CT_-lowest overestimated the severity of the lesion when compared to FFR_CT_ measured within 20 mm of the stenotic lesion in identifying lesion-specific ischemia and false positive results of FFR_CT_-lowest could be effectively reclassified by FFR_CT_ measured within 20 mm of the stenotic lesion. This might be associated with the gradual decrease in FFR_CT_ from proximal to distal along the vessel even without focal stenosis, which is more significant with FFR_CT_ than with invasive FFR due to pressure loss by frictional losses according to Poiseulle’s equation [16]. Taken together, FFR_CT_-1 cm and FFR_CT_-lowest both are not optimal site for FFR_CT_ measurement and cannot be used as FFR_CT_ result in clinical decision-making.

CCTA can overestimate the severity of stenosis in CAD. It has been reported that less than a half of severe coronary artery disease diagnosed by CCTA can really result in lesion-specific ischemia [26]. This raised the concern that the widespread use of CCTA may encourage unnecessary ICA [27]. In our study, the AUC of CCTA (DS ≥ 50%) alone had only a moderate diagnostic performance (AUC = 0.576) for identifying lesion-specific ischemia, which was lower than that of FFR_CT_-1 cm, FFR_CT_-2 cm, FFR_CT_-3 cm, and FFR_CT_-lowest. When CCTA was combined with either FFR_CT_-1 cm, FFR_CT_-2 cm, FFR_CT_-3 cm, or FFR_CT_-lowest, its AUC was improved in identifying lesion-specific ischemia. These results suggest that the addition of FFR_CT_ can improve the diagnostic performance of CCTA in identifying lesion-specific ischemia and may reduce unnecessary ICA, thereby enhance its role as a gatekeeper for ICA.

Our study has some limitations. First, it is a retrospective study from a single center and the sample size was not large. It has selection bias inherent in a retrospective study. Second, patients with previous revascularization were excluded from the study. Thus, the validity of FFR_CT_ parameters in these patients needs further investigation. Third, a per-vessel analysis was performed in our study. In some patients, more than one vessel was included for analysis. Fourth, FFR_CT_ can be calculated using a machine learning-based algorithm or a CFD-based algorithm. In our study, only CFD-based algorithm was investigated. Fifth, the long-term effect of FFR_CT_ on the adverse cardiac events was not investigated in this study. Further prospective clinical studies are warranted to validate the impact of FFR_CT_ on the clinical outcome in patients with CAD.

## Conclusion

Our study demonstrates that FFR_CT_ measured at 2 cm distal to the lower border of the target lesion is the optimal measurement site in identifying lesion-specific ischemia in patients with CAD. The addition of FFR_CT_ to CCTA can improve the diagnostic performance in in identifying lesion-specific ischemia. FFR_CT_-2 cm could be used as an alternative imaging biomarker in identifying lesion-specific ischemia. The use of FFR_CT_-2 cm can avoid unnecessary invasive FFR in a patent who has a 30%-90% diameter stenosis but negative FFR_CT_-2 cm. This may aid in the decision-making in patients with CAD. In our study, the invasive FFR was used as a reference standard to assess the diagnostic performances of FFR_CT_ measured at different sites. Whereas the sample size was relatively small. Future multicenter prospective studies are needed to validate the clinical role of FFR_CT_-2 cm.

## Data Availability

The datasets used and analysed during the current study available from the corresponding author on reasonable request.

## Abbreviations

CAB: Gcoronary artery bypass graft surgery
CAD: Coronary artery disease
CCTA: Coronary computed tomography angiography
CFD: Computational fluid dynamics
EF: Ejection fraction
FFR: Fractional flow reserve
FFR_CT_: Computed tomography fractional flow reserve
ICA: Invasive coronary angiography
NYHA: New York Heart Association
PCI: Percutaneous coronary intervention
ROI: Region of interest
SCCT: Society of Cardiovascular Computed Tomography
